# Invariant asymmetry renews the lymphatic vasculature during homeostasis

**DOI:** 10.1186/s12967-016-0964-z

**Published:** 2016-07-11

**Authors:** Alicia L. Connor, Philip M. Kelley, Richard M. Tempero

**Affiliations:** Department of Neurosensory Genetics and Otolaryngology and Head and Neck Surgery, Boys Town National Research Hospital, 555 North 30th Street, Omaha, NE 68131 USA

## Abstract

**Background:**

The lymphatic vasculature regulates tissue physiology and immunity throughout life. The self renewal mechanism that maintains the lymphatic vasculature during conditions of homeostasis is unknown. The purpose of this study was to investigate the cellular mechanism of lymphatic endothelial cell (LEC) self renewal and lymphatic vessel maintenance.

**Methods:**

Inductive genetic techniques were used to label LECs with tandem dimer tomato (tdT) in adult mice. Two types of studies were performed, those with high dose inductive conditions to label nearly all the lymphatic vessels and studies with low dose inductive conditions to stochastically label individual clones or small populations of LECs. We coupled image guidance techniques and live fluorescence microscopy imaging with lineage tracing to track the fate of entire tdT^+^ cutaneous lymphatic vessels or the behavior of individual or small populations of LECs over 11 months. We tracked the fate of 110 LEC clones and 80 small LEC populations (clusters of 2–7 cells) over 11 months and analyzed their behavior using quantitative techniques.

**Results:**

The results of the high dose inductive studies showed that the lymphatic vessels remained tdT^+^ over 11 months, suggesting passage and expression of the tdT transgene from LEC precursors to progenies, an intrinsic model of self- renewal. Interestingly, the morphology of tdT^+^ lymphatic vasculature appeared relatively stable without significant remodeling during this time period. By following the behavior of labeled LEC clones or small populations of LECs individually over 11 months, we identified diverse LEC fates of proliferation, quiescence, and extinction. Quantitative analysis of this data revealed that the average lymphatic endothelial clone or small population remained stable in size despite diverse individual fates.

**Conclusion:**

The results of these studies support a mechanism of invariant asymmetry to self renew the lymphatic vasculature during homeostasis. These original findings raise important questions related to the plasticity and self renewal properties that maintain the lymphatic vasculature during life.

**Electronic supplementary material:**

The online version of this article (doi:10.1186/s12967-016-0964-z) contains supplementary material, which is available to authorized users.

## Background

The lymphatic vasculature is required for life and it regulates essential aspects of physiology and immunity during conditions of homeostasis and disease throughout life [1, 2]. While it is well known that the lymphatic vasculature proliferates and remodels extensively to meet the demands of disease and wound repair conditions [3, 4], there are no proposed conceptual self renewal models to explain how the lymphatic vasculature is maintained over the lifetime of an animal. To maintain homeostasis of any organ in the post-natal period, loss of cells must be balanced by a proliferation of newly generated cells. Given the importance of the lymphatic system, it seemed reasonable to consider the existence of a self-renewal program. We hypothesized that ‘new’ lymphatic endothelial cells (LECs) would replace LECs regularly to maintain the lymphatic vasculature. We considered mechanisms, such as proliferating neighboring LECs, or alternatively progenitor cells, as sources of the ‘new’ LECs. Although controversial, there are several lines of evidence that non-venous derived progenitor cells contribute to embryologic and pathologic lymphangiogenesis [5–7].

To investigate this hypothesis, we used inducible cre-lox based genetic lableing and intravital microscopy approaches to directly visualize fluorescently labeled lymphatic vessels and individual LEC clones or small populations of LECs during conditions of homeostasis. Mice transgenic for the hormone regulated Cre recombinase driven by the LYVE-1 promoter (Lyve1CreERT2) were developed in our laboratory [8]. The LYVE-1 promoter restricted Cre activity spatially to the LECs and a small population of LYVE-1^+^ macrophages (discussed in greater detail below). The Cre-ERT2s generation construct is a fusion protein that is sensitive to low levels of tamoxifen and displays markedly less activation by endogenous estrogens [9]. Lyve1CreERT2 mice were bred to mice transgenic for the tandem dimer tomato (tdT) fluorescence protein that contains an upstream stop codon flanked by lox P sites [10], to produce Lyve1CreERT2^tdT^ mice. Cre activity was regulated temporally by administering 4-hydroxytamoxifen (4-OHT). By modifying the 4-OHT dose and schedule, we were able to induce tdT in nearly the entire cutaneous lymphatic vasculature or in individual LEC clones or small populations of LECs stochastically in Lyve1CreERT2^tdT^ mice. We adapted this labeling strategy from Mascre et al. [11].

We used the Lyve1CreERT2^tdT^ mice as an in vivo platform for lineage tracing techniques. Lineage tracing is the marking and subsequent identification of all progeny from a founder or progenitor cell [12]. One important principle of labeling cells by inductive genetic recombination is that the inductive agent is administered transiently rather than continuously at the start of the lineage tracing experiment. Transient Cre activation excises the stop codon in the reporter transgene such that these cells express the modified transgene and pass this modified constitutively expressed transgene to all progeny. This indelible labeling enables the detection and tracking of fluorescent founder cells and all progeny. We used this system to investigate the overall remodeling of the lymphatic vasculature and to quantify individual LEC behavior longitudinally over 11 months using intravital microscopy.

## Methods

### Mouse strains

All animal protocols were approved by Boys Town National Research Hospital Institutional Animal Care and Use Committee Institutional Review Board in accordance with NIH guidelines (Protocol #15–01). The development of the Lyve1CreERT2^tdT^ mouse strain has been described in detail [8].

### 4-OHT induction protocols

In previous studies [8], we modified the 4-OHT dose and administration schedule such that 1 mg 4-OHT suspended in sunflower oil administered to 8–10 week old male and female Lyve1CreERT2^tdT^ mice by intraperitoneal route on two consecutive days induced tdT expression in virtually all LECs. This 4-OHT dose and schedule was used for the high dose studies. 0.25 mg 4-OHT administered by intraperitoneal route induced tdT expression in small or clonal LEC populations. 0.25 mg 4-OHT dose was used for the low dose studies designed to follow clone or small LEC populations.

### Microscope image acquisition

All images were acquired at ambient temperature: approximately 23 °C.

### Live imaging

Live imaging was performed on sedated Lyve1CreERT^tdT^ mice. The pinna was depilated and placed between glass slides, and the mouse was positioned laterally. Images were obtained using a Leica MZ10F Fluo III microscope using a Leica Planap 1.0X objective and a Leica DFC310FX camera (acquisition software: LAS version 4.0.0.8777) or a Ziess Axio Zoom.V16 and a Zeiss Plan-NeofluarZ 1.0 × 0.25 na objective and a Zeiss AxioCam MRm camera (acquisition software: Zeiss Zen 2012, blue edition, version 1.1.1.0). To acquire the clonal and small population LEC data, we used low magnification light microscopy to identify the major blood vessels within the pinna. These large stable structures were used to develop a vascular map of the pinna. Using this information to provide image guidance and the identical power of magnification, we were able to visualize the same fields of interest using light and fluorescent microscopy.

### Tissue staining and antibodies

To visualize lymphatic vessels within their microenvironment and study specific features that were identified during live imaging, pinnas were fixed in 1 % paraformaldehyde in PBS pH 7.4 and labeled as previously described using whole mount technique [13]. Whole mount mouse cornea and pinna was stained with antibodies to LYVE-1 (11-034, AngioBio, Del Mar, CA), DAPI (Sigma–Aldrich) and the appropriate secondary antibody: using the fluorochromes Alexa488, (ThermoFisher Scientific) and DyLight488 (Jackson ImmunoResearch Laboratories, West Grove, PA). Fixed and labeled whole mounts were mounted in Vectashield H-1000 (Vector Laboratories, Burlingame, CA).

### Epifluorescence microscopy

Epifluorescent images were acquired using a Zeiss Axio-Imager.A1 and an EC Plan-Neofluar 10 × 0.3 na objective and a Diagnostic Instruments SpotFlex model 15.2 64 Mp Shifting Pixel camera (acquisition software: SPOT windows version 4.6 or 5.1).

### Confocal microscopy

Confocal images were acquired on either a Leica TCS SP8 MP (Creighton University Integrated Biomedical Imaging Facility) using either a HC PL Apochromat 20x 0.75 na objective or a HC PL Apochromat 40 × 1.3 na oil objective (acquisition software: Leica LAS AF version 3.2.1.9702, 12 bit) or a Zeiss AxioObserver LSM 710 (University of Nebraska Medical School Confocal Laser Scanning Microscope Core Facility) using either a Plan-Apochromat 20 × 0.8 na objective or an EC Plan-Neofluar 40 × 1.30 na oil objective (acquisition software: Zeiss Zen 2011).

### Processing software

Channel separation and maximum intensity projections were done in the FIGI version of ImageJ (1.47v) or in the respective confocal acquisition software. Figures were prepared from original images in Adobe Photoshop.

## Results

### Development of Lyve1CreERT2^tdT^ mice

We developed an inductive genetic strategy to express the fluorescent reporter tdT protein in LECs in adult mice. This model was characterized in greater detail in a recent manuscript [8]. Briefly, to target LECs, we generated Lyve1CreERT2 transgenic mice carrying a Cre recombinase-estrogen receptor element driven by BAC construct containing the LYVE-1 promoter. The Lyve1CreERT2 strain was crossed with the floxed stop tdT fluorescence reporter strain, B6.Cg-Gt(ROSA)26Sor tm14(CAG − tdT)Hz/J. This reporter strain expresses tdT fluorescence in the presence of activated Cre and has been used in several inductive Cre transgenic systems and lineage studies [10].

### Lymphatic vasculature was stable during homeostatic conditions

**Fig. 1:**
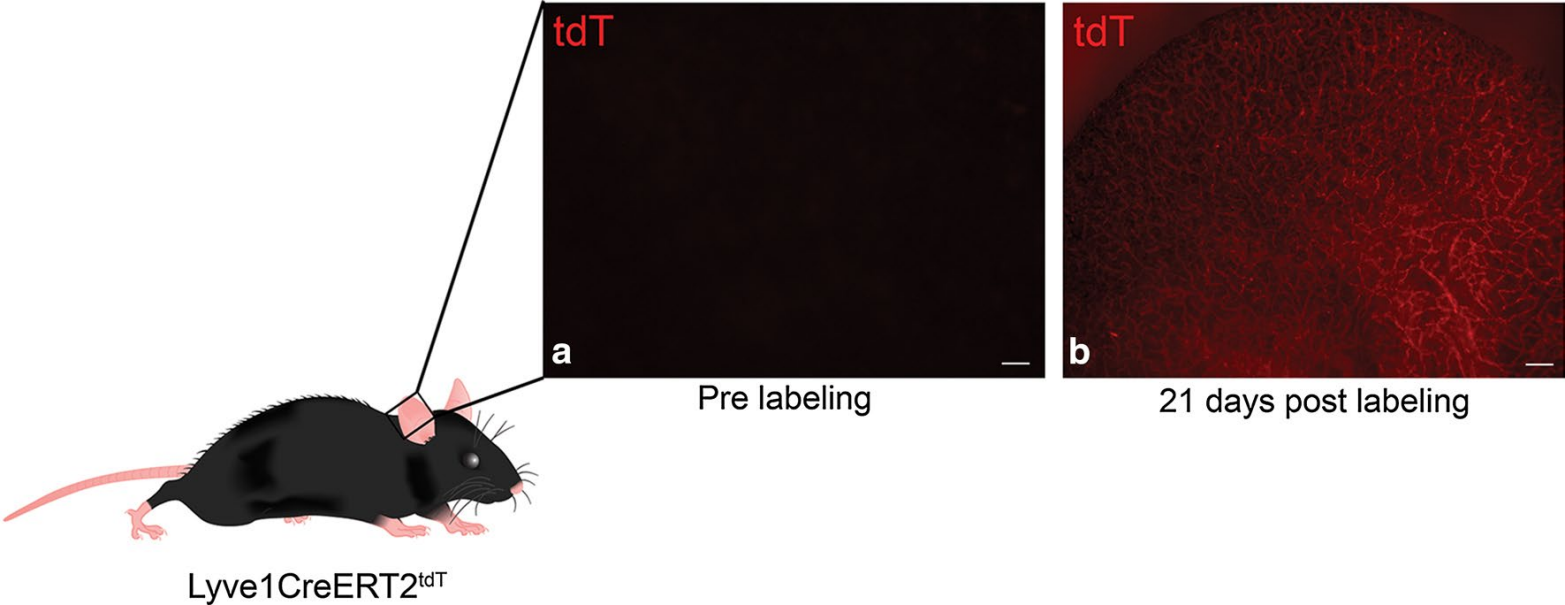
Visualizing cutaneous lymphatic vessels in Lyve1CreERT2^tdT^ mice using live imaging. Low power live imaging epifluorescent microscopy of sedated Lyve1CreERT2^tdT^ pinna reveals no detectable tdT labeling within the pinna (**a**). Using similar microscopy techniques, tdT^+^ lymphatic vessels were detected in the same animal 21 days following 4-OHT administration (**b**). The size standard is 0.5 mm

We designed studies to investigate the remodeling of the cutaneous lymphatic vasculature longitudinally in Lyve1CreERT2^tdT^ mice. We used bright field and fluorescence microscopy techniques to obtain images from the pinna of sedated Lyve1CreERT2^tdT^ mice. Prior to 4-OHT administration, there was no detectable tdT within the Lyve1CreERT2^tdT^ pinna. 3 weeks after induction, tdT fluorescence was readily detected using live imaging fluorescence microscopy (Fig. 1). The entire lymphatic vasculature within the animal was labeled with tdT; however, we elected to investigate the pinna as this tissue was accessible and provided the opportunity to obtain images using intravital microscopy with reasonable resolution when the pinna was gently positioned between two glass slides. Animal movement as a result of the work of breathing and cardiac contractility created continuous movement in sedated mice that made it difficult to obtain adequate images from other cutaneous regions such as the cervical or thoracic regions. The pinna contained two lymphatic plexuses, each deep to overlying epithelium. Thus, the image in Fig. 1b is a composite of two lymphatic plexuses.

**Fig. 2:**
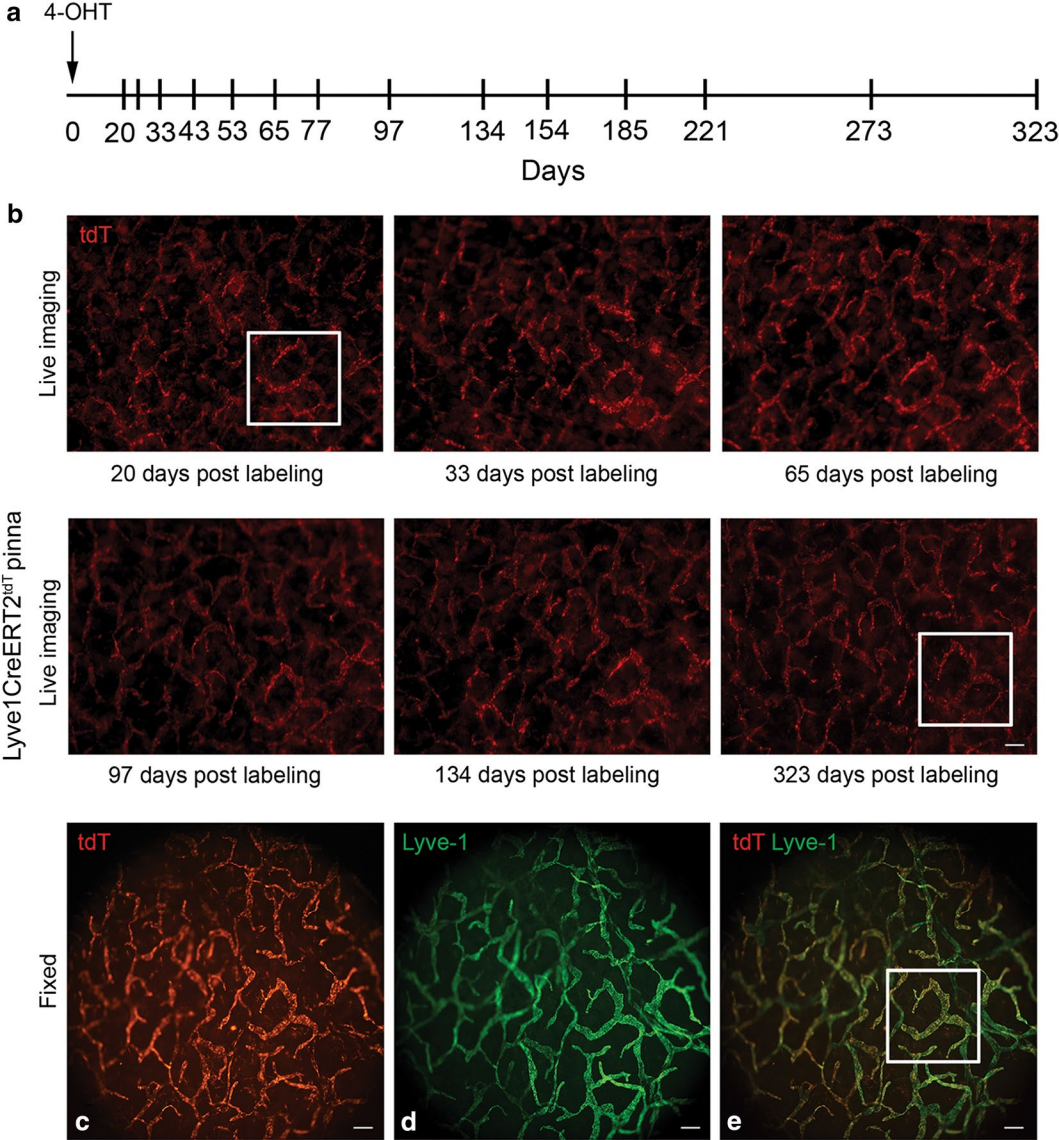
Little remodeling was detected in the lymphatic vessel network in the Lyve1CreERT2^tdT^ pinna. The experimental strategy is illustrated in **a**. The *vertical lines* represent points in time when live imaging was performed. 4-OHT was administered to Lyve1CreERT2^tdT^ mice to label LECs within the pinna. Live imaging epifluorescent microscopy of 2 sedated Lyve1CreERT2^tdT^ mice was performed, first every 3 or 4 days, then with increasing intervals, at 3 low magnification fields within the right pinna to directly visualize the tdT^+^ lymphatic vessels. The morphology of the tdT^+^ lymphatic vessel network within the microenvironment was detectable and relatively stable over the course of 323 days post-labeling (*panel B*). The overall morphology of many of the lymphatic vessels was similar over the course of the study (**b**-inset). At the conclusion of this study, the pinnas were labeled with antibodies to LYVE-1 and evaluated using immunofluorescent microscopy techniques. Low power epifluorescent microscopy showed that most of the tdT^+^ cells were LYVE-1^+^ LECs comprising lymphatic vessels (**c–e**). The results are representative of 2 different Lyve1Cre-ERT2^tdT^ mice. The size standards are 100 µm

To investigate the overall remodeling of the cutaneous lymphatic vasculature during homeostasis, high dose 4-OHT was administered to Lyve1CreERT2^tdT^ mice (n = 2) to transiently activate Cre and induce tdT fluorescence in cutaneous LECs. In these same mice, live imaging was performed initially weekly and then monthly or every other month to visualize the cutaneous tdT^+^ lymphatic vessels within the pinna. The general design of this experiment is shown in Fig. 2a. In sedated Lyve1CreERT2^tdT^ mice, we used guidance techniques based on the identification of prominent blood vessels and their branching structures to locate the microscopy fields of interest in bright field conditions. These relatively large blood vessels coursing within pinna were easy to identify and were stable over the duration of our studies Additional file 1: Figure S1. We captured the endogenous tdT fluorescence signal in three fields of the pinna in sedated Lyve1CreERT2^tdT^ mice initially about every 7 days for 6 weeks. Over the first 6 weeks, we were unable to detect lymphatic vessel growth, regression, or significant changes in overall lymphatic vessel morphology. Based on the lack of detectable changes we increased the observation interval. The overall morphology of the tdT^+^ lymphatic vessel network within the cutaneous microenvironment appeared relatively stable over the course of 323 days. Many of the lymphatic vessels had similar morphology throughout the duration of the study (Fig. 2b panel and e-inset).

At the conclusion of this study, the pinnas were labeled with antibodies to LYVE-1 and evaluated using immunofluorescent microscopy techniques. Most of the LYVE-1^+^ LECs expressed tdT endogenously (Fig. 2c–e) and there were no detectable tdT^−^ LYVE-1^+^ lymphatic vessels in these fields. We considered these observations from a self-renewal standpoint. The fixed pinna was analyzed 11 months following labeling and tdT was widely expressed in the LECs comprising the lymphatic vessels. This data was consistent with a model of intrinsic self renewal. Proliferation of tdT^+^ LECs would predictably generate tdT^+^ LEC progeny, as the modified tdT transgene is passed to all progeny [8] Such a mechanism would generate experimental results similar to what we observed, durable tdT expression within the LECs comprising the vessels.

These observations did not support the proliferation or the incorporation of an unlabeled progenitor population as a major mechanism of self renewal. This type of repopulation process would predictably result in regions of tdT^−^ lymphatic vessels, a finding we did not observe. This is an important consideration as non venous and bone marrow derived precursors have been suggested to contribute to lymphatic vessel embryogenesis and pathologic lymphangiogenesis. These results compelled us to adapt our experimental strategy to track and quantify the behavior of individual tdT^+^ LEC clones over time during homeostasis.

### Cell specificity of tdT^+^ expression in Lyve1CreERT2^tdT^ mice

**Fig. 3:**
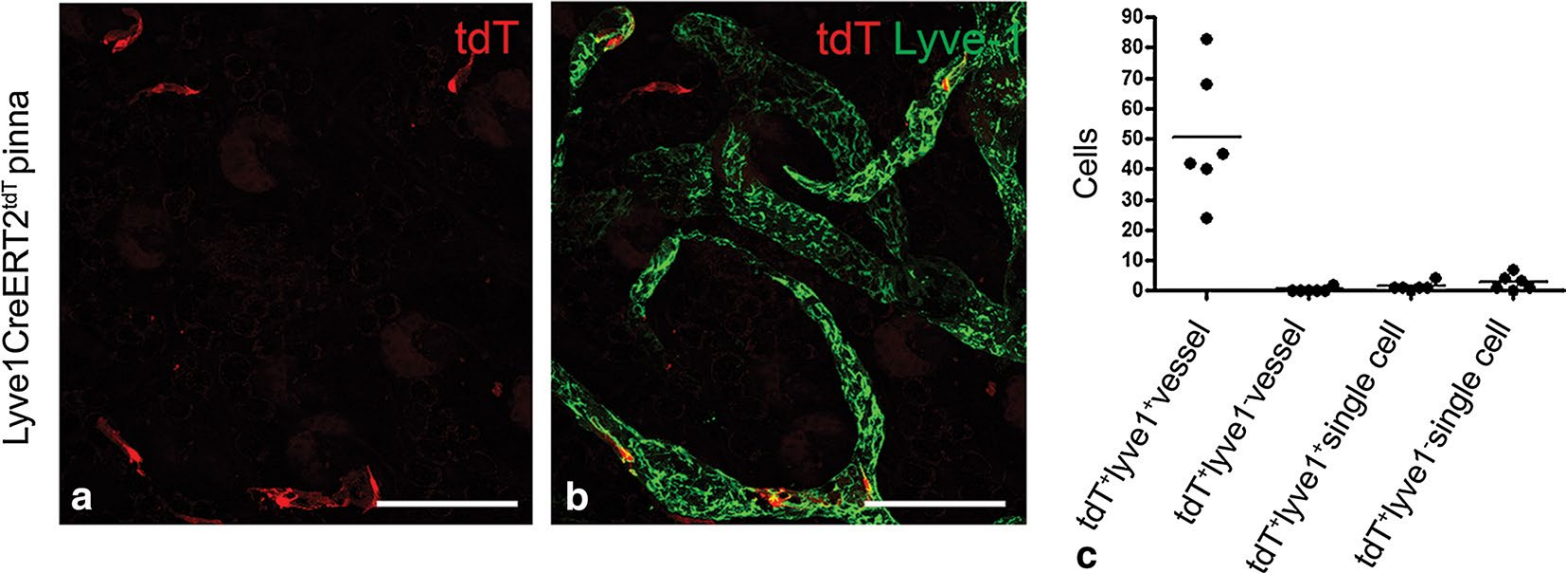
Expression of tdT fluorescence in Lyve1CreERT2^tdT^ pinna following low dose 4-OHT administration. Three weeks after 0.25 mg 4-OHT was administered to 6 Lyve1CreERT2^tdT^ mice, the pinnas were harvested and labeled with antibodies to LYVE-1. Maximum intensity projection images obtained using confocal microscopy were used to determined the targeting specificity of the transgene. 2 images from each mouse pinna were analyzed. Using images similar to the image shown in **a**, tdT^+^ cells were quantified. Using images similar to that shown in **b**, it was determined whether the tdT^+^ cells were LYVE-1^+^ or LYVE-1^–^ and whether these cells were a constituent of a lymphatic vessel. Most of the tdT^+^ cells were LYVE-1^+^ and were constituents of lymphatic vessels (93 %). Some of the tdT^+^ cells were LYVE-1^+^ or LYVE-1^−^ and physically separated from a lymphatic vessel (7 %) (**b,** **c**). The histogram is data pooled from fields obtained from 6 similarly treated Lyve1CreERT2^tdT^ mice. The size standards are 50 µm

To track LEC clone fate, we developed low 4-OHT induction conditions such that tdT fluorescence was expressed in individual cells in the Lyve1CreERT2^tdT^ pinna. As LYVE-1 is expressed in LECs and a population of macrophages [14], we expected both populations to express tdT following 4-OHT administration. Three weeks after 0.25 mg 4-OHT was administered to transiently active Cre in Lyve1CreERT2^tdT^ mice, pinnas were harvested and labeled with antibodies to LYVE-1 to detect LECs. 93 % of the tdT^+^ cells were LYVE-1^+^ LECs integrated stochastically in cutaneous lymphatic vessels. The remaining 7 % were single tdT^+^ cells that were not constituents of a lymphatic vessel (Fig. 3a–c). This data showed that under the low 4-OHT conditions, 93 % of the tdT^+^ cells were LYVE-1^+^ LECs and 7 % were non-LEC, presumably macrophage or other single cells.

### tdT^+^ LEC clone fates of extinction, quiescence, and proliferation during conditions of homeostasis

**Fig. 4:**
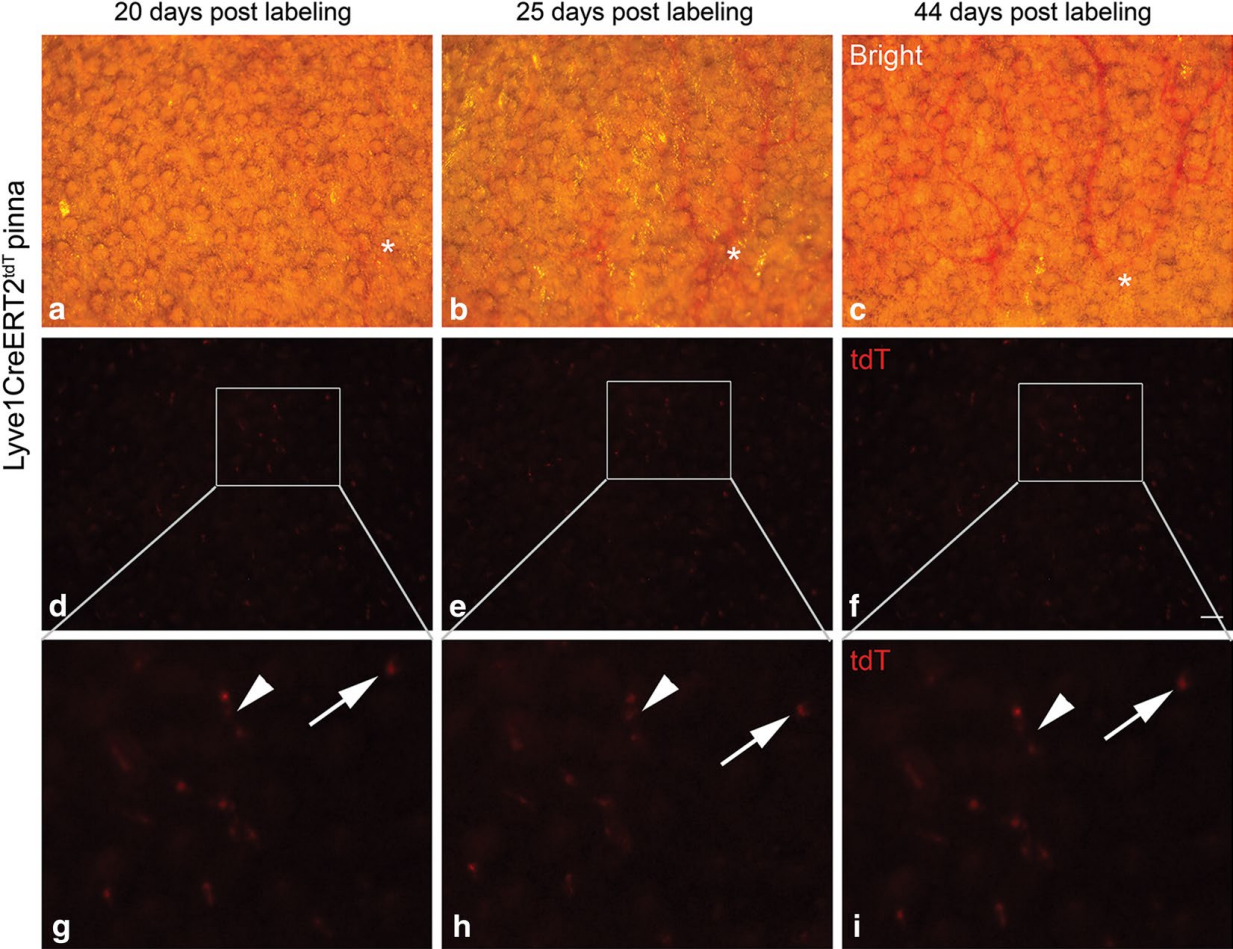
Live imaging methodology for capturing the same fields of interest overtime within the Lyve1CreERT2^tdT^ pinna containing single tdT^+^ clones and small populations. A single 0.25 mg 4-OHT dose was administered to 4 Lyve1CreERT2^tdT^ mice to induce tdT fluorescence stochastically in individual cells. Using low power brightfield microscopy, regions of interest within the pinna were identified based on the morphologic features of large blood vessels (**a**–**c**). An *asterisk* shows a blood vessel branch point as an example. Without adjusting the position of the pinna or the focus, the Texas Red filter was used to detect the endogenous tdT fluorescence (**d**–**f**). The magnified fields show examples of single tdT^+^ clones (*arrow*) and a tdT^+^ small population of 3 cells (*arrowhead*) (**g**–**i**). The tdT^+^ cells were visualized 20 (**g**), 25 (**h**), and 44 (**i**) days following labeling. This methodology was used to identify 110 tdT^+^ clones and 80 tdT^+^ small populations that were tracked over time. The size standard is 100 µm

Low dose 4-OHT was administered to a cohort of 4 Lyve1CreERT2^tdT^ mice to induce tdT fluorescence in individual cells. We used guidance techniques based on blood vessel position and morphology visualized in the bright field to identify 3 regions in the pinna of sedated Lyve1CreERT2^tdT^ mice. An example of one region is shown 20, 25, and 44 days post labeling (Fig. 4a–c). Without changing the position of the mouse, the texas red filter was used to visualize the tdT fluorescence in the pinna (Fig. 4d–f). These techniques were performed serially over the course of 323 days. In the images obtained using fluorescence microscopy single tdT^+^ cells and clusters of tdT^+^ cells were detected (Fig. 4g–i). We identified 110 tdT^+^ clones and 80 tdT^+^ small populations using these techniques and tracked the individual fate of these different populations over time.

### Population homeostasis despite different LEC clone fates

**Fig. 5:**
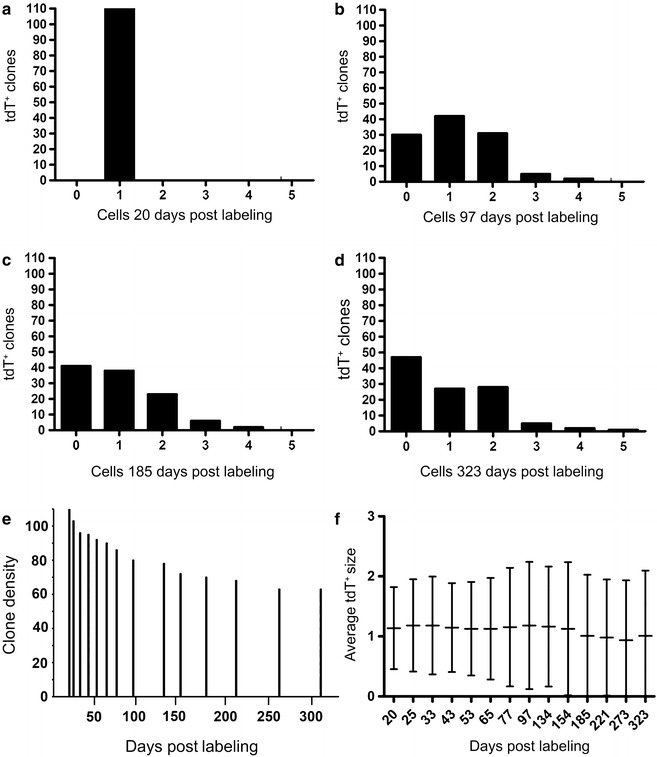
The fate of 110 tdT^+^ clones was diverse despite a balanced population size. 110 tdT^+^ clones were identified 20 days after labeling (**a**), Histograms **b**–**d** show individual tdT^+^ clone fates at the indicated times post-labeling and the evolution of this process over 323 days. For example, at 323 post labeling, 45 clones were undetectable, 25 remained 1 cell, and 40 expanded to 2–5 cells. Analysis of the population showed that the survival or clone density decreased and stabilized over 323 days (**e**). Quantitative analysis showed that the average tdT^+^ clone size (calculated by dividing the number of total starting tdT^+^ clones (110)/the number of tdT^+^ cells at a given point in time) remained approximately one over 323 days (**f**). This was pooled data from 4 Lyve1CreERT2^tdT^ mice

The fate of 110 tdT^+^ clones changed considerably over 323 days. tdT^+^ clonal extinction, quiescence, or proliferation were visualized and quantified. At the conclusion of the study 45 clones were no longer detectable (extinct). 25 clones remained as one cell (quiescence), and some clones had proliferated to become clusters of 2–5 tdT^+^ cells (Fig. 5a–d). Analysis of the individual tdT^+^ clone fates showed heterogeneous behavior over 323 days. To study the behavior of tdT^+^ clones as a population we used quantitative analysis. The function of tdT^+^ clones surviving (clone density) over time produced a death rate of 0.3 tdT^+^ clone/day (Fig. 5e). The average tdT^+^ clone size (calculated by dividing the number of total starting tdT^+^ clones (110)/the number of tdT^+^ cells at a given point in time) remained approximately 1 over 323 days (Fig. 5f). Because the average clone size was 1, we concluded that the clone proliferation and death rates were approximately equal, 0.3 tdT^+^ clone/day.

### LEC population homeostasis despite different fates of proliferation, extinction, and quiescence in small populations of tdT^+^ LECs

**Fig. 6:**
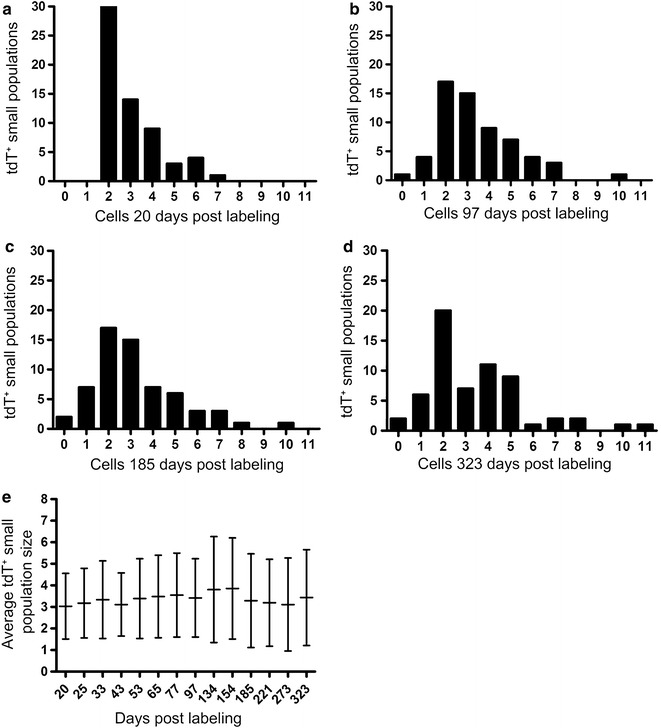
The fate of 80 tdT^+^ small populations was heterogeneous despite a balanced population size. The starting small population (clusters of 2–7 cells) distribution at 20 days post labeling is shown in **a**. Histograms **b**–**d** show the individual fate of small populations at the indicated times post-labeling and the evolution of this process over 323 days. For example, at 20 days post labeling, there were 30 small populations of 2 tdT^+^ cells, decreasing overtime to 20 small populations of 2 tdT^+^ cells after 323 days. Despite diverse small LEC population fates, quantitative analysis showed that the average tdT^+^ small LEC population was 3–3.5 tdT^+^ cells over 323 days (**e**). This was pooled data from 4 Lyve1CreERT2^tdT^ mice

We tested this concept in greater detail by quantifying the behavior of small populations of contiguous tdT^+^ LECs in the pinna of Lyve1CreERT2^tdT^ mice. Using the same Lyve1CreERT2^tdT^ cohorts and live imaging microscopy fields, we tracked the behavior of contiguous small populations of tdT^+^ LECs. 80 individual contiguous groups of 2–7 tdT^+^ cells were tracked and quantified for 323 days. There was a range of tdT^+^ small populations at 20 days post labeling (Fig. 6a). Overtime, this distribution changed considerably (Fig. 6b–d). For example, at 20 days post labeling there were 30 small populations of 2 tdT^+^ cells that decreased overtime to 20 small populations of 2 tdT^+^ cells after 323 days. Despite heterogenous small LEC population behavior, quantitative analysis showed that the average tdT^+^ small LEC population was relatively constant at 3–3.5 tdT^+^ cells over 323 days (Fig. 6e). These findings are consistent with the tdT^+^ clone data.

## Discussion

We tracked and quantified the behavior of individual LEC clones in vivo during homeostasis and showed heterogeneous LEC behavior that was consistent with an invariant asymmetry model of self renewal. This is the first report to explore the cellular mechanisms of lymphatic vessel self renewal.

The use of inductive genetic recombination and lineage tracing has generated significant advances in our understanding of the mechanisms that regulate epithelial [11, 15–17] and tumor [15, 18] cell and population dynamics during conditions of homeostasis and injury. Direct observation of labeled cells in vivo during lineage tracing facilitates the identification of founder or progenitor cells and the interpretation of cell behavior or fate over time [12]. Thus, the direct observation techniques used here, allowed the identification of founding LECs and the analysis of individual LEC behavior. These direct observation techniques have limitations. For example, it is not possible to directly observe biologic events over time and simultaneously harvest tissue to obtain histologic ‘snapshot’ data of more classic indicators of proliferation without disrupting the experimental design.

We observed minimal lymphatic vessel remodeling over the length of the study. This was surprising, as we anticipated detecting more dynamic lymphatic vessel remodeling within the cutaneous microenvironment. The persistence of the tdT label in the LECs prompted us to consider several mechanisms of self renewal. This finding raised the question of whether all of the founding tdT^+^ LECs persisted throughout the entire study. We considered this to be unlikely. Alternatively, we considered an intrinsic mechanism of self renewal, such that some of the founding tdT^+^ LECs proliferated and passed the modified tdT transgene to LEC progeny. Recently, a non-venous origin of the dermal lymphatic vasculature was described as a mechanism of lymphatic vessel development in the mouse lumbar and thoracic regions during embryogenesis [6]. We considered whether a similar extrinsic mechanism would self renew the lymphatic vasculature in the post-natal period. The replacement of tdT^+^ LECS with an unlabeled LEC progenitor, would predictably result in a loss of tdT^+^ LECs and the accumulation of tdT^−^ lymphatic vessels over time. We did not observe such findings.

We explored the possibility of an intrinsic self renewal mechanism by tracking and quantifying individual tdT^+^ LEC behavior. By modifying the induction scheme, we were able to label single cell clones and small populations of LECs with tdT. Using the blood vasculature as an internal guide, we were able to reproducibly capture serial images using fluorescent microscopy. We tracked the fate of these populations over 11 months and investigated clone fate by applying quantitative analysis to study the behavior of these populations. We visualized the loss of detection, the persistence, and the expansion of single tdT^+^ cells and small tdT^+^ populations that remained as assemblages changing slowly over the course of months. Endothelial cell shuffling could explain these observations; however, we do not favor this interpretation. Endothelial cell rearrangement has been suggested as a highly dynamic cellular mechanism of angiogenesis (cells moving in real time). This model is based upon results of studies conducted primarily in vitro or ex vivo [19, 20]. The results of recently published work showed that LECs do not rearrange or shuffle in vivo during corneal lymphangiogenesis [8]. In addition, we found it difficult to reconcile the shuffling model with the results presented here. For example, the spatial position of most of the tdT^+^ cells was highly conserved over time (Fig. 4; Additional file 1: Figure S1) and 30 % of the tdT^+^ quiescence LEC clones were detected in the exact same tissue position over 11 months. We acknowledge that lymphatic endothelial rearrangement may occur slowly at a rate that could not be detected experimentally.

Quantitative analysis of clonal fate data has provided new insights into the homeostatic mechanisms of cycling or proliferative tissue such as the mammalian intestine and epidermis [21, 22]; however, far less is known about other mammalian organ systems. In part, these types of studies are changing the concepts of stem cells and mechanisms that regulate tissue maintenance. The ability to track the fate of proliferating cells in vivo with genetic lineage tracing has lead to a rebirth of the concepts that govern the basic principles that establish the proliferative hierarchy and capacity in adult tissue. Multiple lines of evidence have revealed a population asymmetry in tissue with high proliferative demands in which the balance of proliferation and differentiation is regulated at the level of the stem cell population [16, 23]. The hallmark of such population asymmetry is that the average clone size increases and clonal heterogeneity diminishes over time [21, 23]. There are examples of invariant asymmetry self renewal where one stem cell gives rise to a stem cell and a differentiated cell in invertebrates [24] and satellite muscle cells [25], resulting in a mosaic of ‘clonal units’ responsible for tissue homeostasis. The hallmarks of invariant asymmetry are a stable clone size and a fraction of surviving clones over time. We identified both of these findings in the lymphatic vasculature during homeostasis.

Here, we show that the fates of individual LECs are diverse; however, the population is balanced. Quantitative analysis revealed a plateau of the LEC clone survival and the constant average LEC clone size of 1. One limitation of this study is that the targeting transgene labeled LECs and macrophages. Approximately 7 % of the tdT^+^ cells were macrophage (Fig. 3). Because of technical reasons, largely tissue orientation, it was not possible to orient and positively identify single antibody stained cells within the pinna. This made it difficult to prove conclusively that the tracked tdT^+^ cells were LECs. Although the macrophages comprised 7 % of the tdT cells, our interpretation of the data remains unchanged.

## Conclusions

**Fig. 7:**
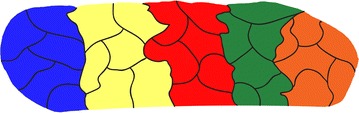
Cartoon showing a multicolor model of lymphatic endothelial clonal subunits. For schematic purposes, a mosaic of lymphatic clonal subunits is shown comprised of individual LECs, each represented by a *different color*. Lymphatic endothelial clonal evolution conforming to a model of invariant asymmetry will predictably result in a mosaic of lymphatic endothelial subunits

The results of this study show that LEC self renewal and lymphatic endothelial clonal units are major mechanisms of lymphatic vessel homeostasis. The data suggests that the peripheral lymphatic vasculature is a slow cycling organ system during homeostasis. The results are consistent with an invariant asymmetry model of self renewal resulting in a mosaic of ‘lymphatic endothelial units’ (Fig. 7). Understanding how these lymphatic clonal units regulate lymphatic vessel growth and remodeling during homeostasis and disease conditions may provide useful therapeutic concepts and targets to contract or expand the lymphatic vasculature.

## Additional file

10.1186/s12967-016-0964-z Live imaging methodology for capturing the same fields of interest overtime within the pinna. Low power brightfield microscopy was used to visualize the major blood vessels within the pinna (A). A blood vessel is shown with an arrowhead and a blood vessel branch is show with an arrow. Ease to identify vascular features were used as a guidance tool to reproducibly visualize the same anatomic field of interest over time in sedated Lyve1CreERT2^tdT^ mice (A-inset). By maintaining the position of the pinna and the focus, epifluorescent microscopy using the Texas Red filter was used to detect the endogenous tdT fluorescence (B). The techniques were used overtime to visualize the tdT^+^ cells with the pinna (C and D). Over a one month interval the spatial distribution of the tdT^+^ cells appeared relatively stable, although subtle changes can be detected with close inspection (B and D).

## Abbreviations

LEC: lymphatic endothelial cell
4-OHT: 4-hydroxytamoxifen
tdT: tandem dimer tomato
