# Supplementary figures and images for: Invariant asymmetry renews the lymphatic vasculature during homeostasis
Source: J Transl Med. 2016 Jul 11;14:209. doi: 10.1186/s12967-016-0964-z (PMC4940917; doi:10.1186/s12967-016-0964-z)

Lyve1CreERT2<sup>tdT</sup> pinna

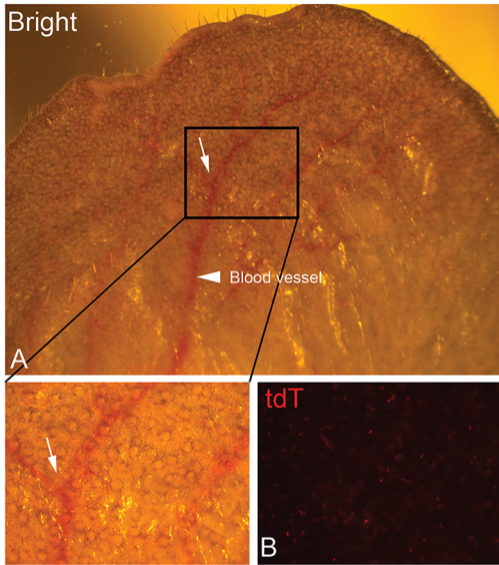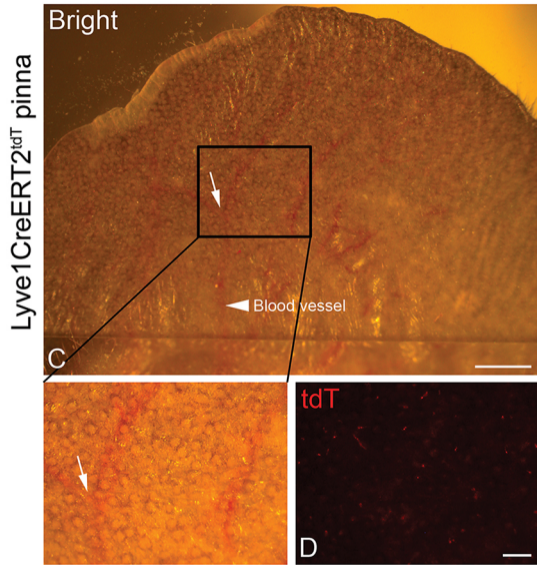

Supplement: Supplementary file 1 — 10.1186/s12967-016-0964-z Live imaging methodology for capturing the same fields of interest overtime within the pinna. Low power brightfield microscopy was used to visualize the major blood vessels within the pinna (A). A blood vessel is shown with an arrowhead and a blood vessel branch is show with an arrow. Ease to identify vascular features were used as a guidance tool to reproducibly visualize the same anatomic field of interest over time in sedated Lyve1CreERT2tdT mice (A-inset). By maintaining the position of the pinna and the focus, epifluorescent microscopy using the Texas Red filter was used to detect the endogenous tdT fluorescence (B). The techniques were used overtime to visualize the tdT+ cells with the pinna (C and D). Over a one month interval the spatial distribution of the tdT+ cells appeared relatively stable, although subtle changes can be detected with close inspection (B and D). [file 12967_2016_964_MOESM1_ESM.pdf]
